# A Case of Definitive Therapy for Localised Prostate Cancer: Report of a Urological Nightmare

**DOI:** 10.1155/2012/632419

**Published:** 2011-09-15

**Authors:** Andreas Sommerhuber, Verena Traxlmayr, Wolfgang Loidl

**Affiliations:** Department of Urology, St. Vincent's Hospital of Charity, 4010 Linz, Austria

## Abstract

Radical prostatectomy, external beam radiotherapy and permanent brachytherapy are the most common treatment options for nonmetastatic localised adenocarcinoma of the prostate (PCa). Accurate pretherapeutic clinical staging is difficult, the number of positive cores after biopsy does not imperatively represent the extension of the cancer. Furthermore postoperative upgrading in Gleason score is frequently observed. Even in a localised setting a certain amount of patients with organ-confined PCa will develop biochemical progression. In case of a rise in PSA level after radiation the majority of patients will receive androgen deprivation therapy what must be considered as palliative. If local or systemic progressive disease is associated with evolving neuroendocrine differentiation hormonal manipulation is increasingly ineffective; radiotherapy and systemic chemotherapy with a platinum agent and etoposide are recommended. In case of local progression complications such as pelvic pain, gross haematuria, infravesical obstruction and rectal invasion with obstruction and consecutive ileus can possibly occur. In this situation palliative radical surgery is a therapy option especially in the absence of distant metastases. A case with local and later systemic progression after permanent brachytherapy is presented here.

## 1. Case Report

A 65-year-old patient was referred to our urological clinic for a prostate biopsy indicated for a PSA elevation of 4,5 ng/mL. The patient presented without any previous morbidities in his medical or urological history and was entirely asymptomatic. Digital rectal examination (DRE) and transrectal ultrasound (TRUS) of the prostate were normal, the size of the gland was 36 mL. An octant biopsy was conducted in January 2002 by which adenocarcinoma of the prostate (PCa) was diagnosed in 1 out of 8 cores. The lesion was circumscribed with a length below 2 mm and a Gleason grade of 2, WHO grade was 1, all the other seven biopsies were classified as benign prostatic hyperplasia and chronic inflammation. Following discussion of all therapeutical options, the patient decided to undergo permanent brachytherapy with J^125^. 62 seeds with 0,467 mCi/seed were implanted, total activity was 28,95 mCi, and postoperative course was without any complications.

During the early postoperative phase, the patient was free of complaints, there was neither a sign of incontinence nor any stool disorder, even the erectile function was assessed by an IIEF-score of 21. PSA was constantly decreasing to reach its nadir of 0,75 ng/mL 15 months after seed implantation.

21 months after brachytherapy, the first increase of PSA up to 2,6 was observed. Presuming the possibility of a so-called “PSA bouncing” with an episode of prostatitis a cycle of antibiotic therapy with ciprofloxacin over three weeks was administered, after which PSA fell again to a level of 2,11. At the subsequent follow-up examination half a year later, the patient presented with obvious local and systemic progression: PSA rose up to 8,1 ng/mL with a doubling time of three months, and digital rectal examination showed a dense left lobe with a firm node on the contralateral side. At this time, the patient refused restaging and rejected the recommended LHRH agonist therapy. After prophylactic radiation to the breasts with a dose of 1500 cGy antiandrogen monotherapy with bicalutamide 150 mg per day was initiated. Receiving this medication a drop of PSA to 2,4 ng/mL was achieved, the patient was furthermore feeling asymptomatic.

Due to a PSA progress up to 10,6 ng/mL after 15 months of antiandrogen treatment, reevaluation was conducted. While choline PET-CT showed an increased fluorocholine (FCH) metabolism in the right lobe of the prostate with no signs of lymph node or bone metastases, a rebiopsy of the gland yielded a dramatic upstaging and upgrading of the local disease: of 15 cores taken 13 were infiltrated by prostate cancer with Gleason Score 8 (4 + 4), WHO grade was 3. Antiandrogen therapy was stopped; the LHRH agonist leuprorelin acetate was administered. Due to the history of brachytherapy, any form of further external beam radiation was not feasible.

Only two months later, the patient had to be hospitalised again because of gross haematuria and clot retention. A CT scan showed local progression with a large solid tumour dorsal of the right side of the prostate and bladder with a diameter of 6 cm as well as pathologically enlarged lymph nodes in the pelvis up to 2 cm. Due to continuous bleeding under bladder irrigation palliative transurethral resection of the prostate had to be performed, 15 g of fragile tumour tissue were removed. The pathological report confirmed the diagnosis of an adenocarcinoma with a Gleason score of 8, but additionally larger areas with neuroendocrine differentiation were found. 

After a period of the next three weeks, transurethral reintervention for severe haematuria had to be undertaken, and due to persistent bleeding transfusions of several red cell concentrates were necessary. Obstruction of the upper urinary tract led to bilateral nephrostomies. After discharge, several recurrent episodes of bleeding and clot retention occurred. The general health status deteriorated preventing the initiation of systemic chemotherapy. 

**Figure 1:**
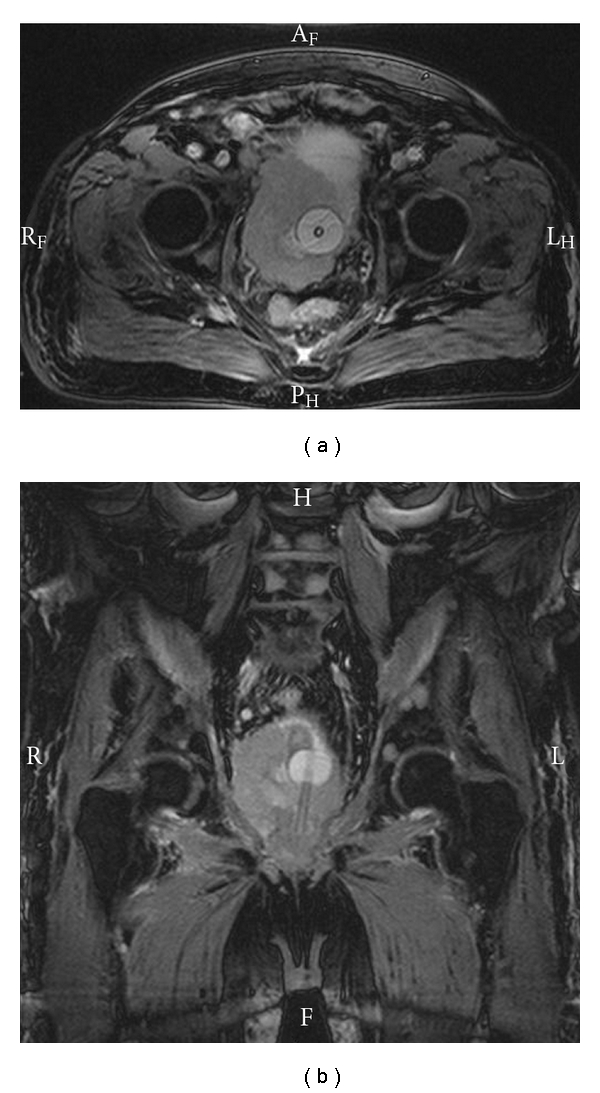
MRI demonstrating a local solid tumour with a diameter of 9 cm infiltrating the trigone and the bladder floor on each side.

Only two months following the previous CT scan, MRI showed a large solid local tumour with a diameter of now 9 cm infiltrating the trigone and the bladder floor on each side (Figure 1). Beneath lymph nodes, up to 3 cm disseminated bone metastases were diagnosed. Four years after the “first definitive” therapy, the patient received a palliative ileal conduit to control local symptoms. With regards to the limited life expectancy, the prostate and bladder remained in situ; recurrent bleeding was controlled by percutaneous transluminal angiography and coil embolization; additionally, instillations with formalin were performed twice. The patient's health status was declining rapidly and three weeks after the last surgery, he died due to dilatation of the right ventricle and pulmonary oedema. Beyond that, autopsy showed extensive pulmonary metastases.

## 2. Discussion

Accurate staging of prostate cancer on the basis of clinical features such as PSA, DRE, and histology of biopsy is difficult. The number of positive cores does not imperatively represent the extension of the cancer, furthermore, in about 50% an upgrading in Gleason score has to be observed after performing radical prostatectomy. Besides, radical prostatectomy and external beam radiotherapy, permanent brachytherapy is one treatment option of nonmetastatic-localised PCa [1]. Even in a localised setting, 10–20% of patients with organ-confined PCa will develop biochemical progression within 5–10 years; if the cancer is locally advanced at the time of treatment, the progression rate increases to up to 30–50% [2–4]. 70% of the patients with PSA relapse after brachytherapy, present with local persistence as the only site of recurrence, depending on the initial tumour stage [2, 5]. In case of a rise in PSA level after radiation therapy the majority of patients will receive androgen deprivation therapy. Since this must be considered as palliative, a reverse strategy is salvage radical prostatectomy (SRP). Heidenreich et al. reported a retrospective series of 55 men with biopsy-proven, locally recurrent PCa after primary radiotherapy. Predictors of organ-confined PCa with negative surgical margins were biopsy Gleason score prior to salvage radical prostatectomy, <50% positive biopsy cores, PSA doubling-time >12 months, and low-dose brachytherapy. SRP was judged as a surgically challenging but effective secondary local treatment with curative intent [2]. Furthermore, studies about cryotherapy, brachytherapy and high-intensity focused ultrasound (HIFU) with partly encouraging results but smaller numbers of patients were published [6]. 

If SRP was not performed after PSA relapse castration resistant PCa with local progression and associated complications such as pelvic pain, gross haematuria, infravesical obstruction, and rectal invasion with obstruction and consecutive ileus can possibly occur in the further course; each of those sequels impairs the patient's well-being significantly. Voiding problems may require lifelong indwelling catheters, obstruction of the ureters often results in long-term placement of nephrostomy tubes or ureteral stents, and despite those measurements renal failure may occur. In this situation, palliative radical surgery is a therapy option especially in the absence of distant metastases. 

Leibovici et al. were looking at the effect of cystoprostatectomy for palliation of symptomatic bladder invasion by prostate cancer. 21 patients had previous local therapy, 17 were primary T4 tumours. During surgery, rectal injuries occurred in 13%, there were no perioperative deaths. In 79% (30/38 patients), local symptoms were relieved permanently after the operation. 3 patients (8%) suffered from persistent pelvic pain, another 3 patients from urinary incontinence, and 1 from on going haematuria. The average interval between surgery and clinical systemic disease was 26 months, median disease specific survival was 31 months [7]. Pfister et al. published a series of 20 patients with locally advanced prostate cancer. 70% had an indwelling catheter due to infravesical obstruction, 10% had nephrostomy tubes inserted, 30% recurrent gross haematuria with the need for blood transfusions, 25% suffered from rectal obstruction with consecutive subileus. 15 patients received cystoprostatectomy, median operation time was 260 min, blood loss was 500 mL. In 80%, durable symptom reduction was achieved, median symptom-free survival added up to 15,3 (6–25) months, median survival up to 20,4 (9–28) months [8].

Neuroendocrine differentiation. The number of neuroendocrine cells increases after puberty until an optimum level that persists between the age of 25 and 54, they represent the third epithelial cell type on normal prostatic tissue in addition to basal and secretory cells. Because of the lack of androgen receptors on these cells in normal and neoplastic prostates, they are androgen-insensitive, so hormonal therapy is not a true option for neuroendocrine prostate cancer [9]. In case of loco-regional disease, surgical resection with or without adjuvant treatment (chemotherapy, radiation) is recommended [10]. For prostate cancer with a Gleason score between 8 and 10, Krauss et al. reported inferior clinical outcomes for patients treated with primary radiotherapy if neuroendocrine differentiation exceeds >1%, 10-year distant metastases rates and cause specific survival were significantly lower. In this paper no differences in outcomes were seen for patients with 0% versus <1% neuroendocrine differentiation [11].

For patients with metastatic-stage disease, systemic chemotherapy with a platinum agent (cisplatin or carboplatin) and etoposide is recommended. However, due to aggressive histological features of neuroendocrine carcinomas response durations are often short [10]. In a study published by Culine et al., 41 patients were treated with a combination of docetaxel and cisplatin. PSA response rate was 48% and clinical benefit was observed in 45% of patients, median survival was 12 months [12]. Stein et al. published a cohort of 30 patients treated with cisplatin-based chemotherapy with or without pelvic radiotherapy. After an initial response, 25 patients succumbed to massive local and/or distant failure [13]. Based on the disappointing results with the established therapy strategies, novel approaches have to be implicated. Somatostatin analogues, serotonin and bombesin antagonists, and cytokines are under investigation [14]. Recently, Sciarra et al. reported a median overall survival superior to the 10-month median survival in patients with hormone refractory disease using a combination of estrogens and somatostatin [15].

The case presented here may have profited from a surgical approach once rapid progression had become obvious. It teaches that palliative radical tumour surgery should be considered in patients with locally advanced disease, especially when symptoms impair the patient's well being. Even in cases with widespread metastases, an effective local symptom control can be achieved for the last months of their lives.
